# Supplementary material for: Change of Hypermucoviscosity in the Development of Tigecycline Resistance in Hypervirulent Klebsiella pneumoniae Sequence Type 23 Strains
Source: Microorganisms. 2020 Oct 10;8(10):1562. doi: 10.3390/microorganisms8101562 (PMC7601201; doi:10.3390/microorganisms8101562)
Supplement: Supplementary file 1 [file microorganisms-08-01562-s001.pdf]

Supplementary Table 1. Primers used in this study.

Primers	Sequence (5' → 3')	Reference*
Primers for sequnecing		
marR-F	AAGAGTGACCCACATCGTACTTT	This study
marR-R	CCACTTAGAGTAACCGGAGCG	
soxR-F	TTTTGTCTGCGGGCGAGTAT	Fang et al., 2015
soxR-R	GCGAGATAATGCGAAAGACA	
acrR-F	GCCTGAGAGCATCAGAACGA	This study
acrR-R	AAATGCCGGAGAATACCGGG	
ramR-F	ATTTTTGCGTTCCGCCGTTG	This study
ramR-R	CCAGCAGTGTTCGGTAAACG	
rpsJ-F	CGCGCCAGAATTTGCGTATAA	This study
rpsJ-R	GCCATCGTTAGCCAGGTCTT	
Primers for qRT-PCR		
Q-ramA-F	ATTTCCGCTCAGGTGATTGA	Roy et al., 2013
Q-ramA-R	GTTGCAGATGCCATTTCGAA	
Q-kpgB-F	GAAGAGGAGATCGCCCATCG	Xu et al., 2016
Q-kpgB-R	GCGTTGTCTCCAGTCCTTGA	
Q-oqxB-F	TCTGATCGTCGAGTTTGCCC	This study
Q-oqxB-R	GCGATAAAGGCGATGGAGGT	
Q-acrB-F	TGAGCGTATCGAGCAGACCT	This study
Q-acrB-R	CCATCAGCCCGGAAAGGTAG	
Q-rpoB-F	CACCTGGGTATGGCTGCAAA	This study
Q-rpoB-R	CAGATCGTAAGCACGCTGGA	
Primers for capsular serotyping		
K1-magA-F	GGTGCTCTTTACATCATTGC	Turton et al., 2008
K1-magA-R	GCAATGGCCATTTCGCTTAG	
K2-wzy-F	GACCCGATATTCATACTTGACAGAG	Turton et al., 2008
K2-wzy-R	CCTGAAGTAAAATCGTAAATAGATGGC	

References

Fang H, Fu Y, Chen Q. Ruan Z, Hua X, Zhou H, Yu Y. Tigecycline susceptibility and the Role of efflux pumps in tigecycline resistance in KPC-producing *Klebsiella pneumoniae*. PLoS ONE 2015; 10: e0119064

Roy S, Datta S, Viswanathan R, Singh AK, Basu S. Tigecycline susceptibility in Klebsiella pneumoniae and *Escherichia coli* causing neonatal septicaemia (2007–10) and role of an efflux pump in tigecycline non-susceptibility. Journal of Antimicrobial Chemotherapy, 2013; 68:1036-1042.

Turton JF, Baklan H, Siu LK, Kaufmann ME, Pitt TL. Evaluation of a multiplex PCR for detection of serotypes K1, K2 and K5 in *Klebsiella* sp. and comparison of isolates within these serotypes. FEMS Microbiology Letters, 2008; 284: 247-252.

Xu H, Zhou Y, Zhai X, Du Z, Wu H, Han Y, Huo C, Chen Y. Emergence and characterization of tigecycline resistance in multidrug-resistant *Klebsiella pneumoniae* isolates from blood samples of patients in intensive care units in northern China. Journal of Medical Microbiology 2016; 65:751-759.
